# Disparities in Preventive Healthcare Utilization & Outcomes Among Sexual Minority and Heterosexual Older Adults

**DOI:** 10.1093/geroni/igaf122.3418

**Published:** 2025-12-31

**Authors:** Julia Fox, Jing Li

**Affiliations:** University of Washington, Seattle, Washington, United States; University of Washington, Seattle, Washington, United States

## Abstract

**Background:**

Research on health disparities among older sexual minority adults (SMA) remains limited. Evidence suggests SMA may have lower preventive healthcare utilization and poorer health outcomes than heterosexual peers, partly due to discomfort in medical settings. However, comprehensive studies outside HIV/AIDS and mental health remain scarce.

**Objective:**

This study examines differences in health outcomes and healthcare utilization between SMA and heterosexual older adults using Health and Retirement Study (HRS) data.

**Methods:**

Data from the 2018–2022 HRS surveys were analyzed to compare self-reported health, chronic conditions, and preventive healthcare utilization (e.g., vaccinations). Descriptive statistics and Pearson’s t-tests assessed group differences, with adjusted models planned.

**Results:**

The sample includes 463 SMA and 8,755 heterosexual older adults (mean age: 70, 60.6% female). SMA were significantly less likely to receive a shingles vaccine and more likely to have missed a flu shot (p < 0.05), with no differences in COVID-19 or pneumonia vaccinations. SMA had higher rates of poor self-rated health, hypertension, diabetes, and cancer (p < 0.05) but lower arthritis prevalence (p < 0.05). No differences were found in chronic pain, osteoporosis, or glaucoma.

**Conclusions:**

SMA experience disparities in some preventive healthcare measures and health outcomes, though patterns vary. These findings highlight the need for targeted interventions and further research to understand underlying factors.

